# Supplementary material for: Diagnosis of neonatal and adult sepsis using a Serum Amyloid A lateral flow test
Source: PLoS One. 2025 Feb 12;20(2):e0314702. doi: 10.1371/journal.pone.0314702 (PMC11819581; doi:10.1371/journal.pone.0314702)
Supplement: S1 Table — Subsequently to acquisition of ethical permission, local administrative clearance was secured from each of the 5 participating Ugandan hospitals. Clinical specimens were collected from patients and data was accessed for research purposes between 7 November 2022 and 18 May 2023. All adult participants in FS provided written informed consent. For NSS, parents or guardians provided written informed consent for neonates. For participants who were unable to read or write, consent was obtained in the presence of a witness who read and explained the ethics statement. Participants confirmed their consent by affixing their thumbprints to the consent forms, which were also countersigned by the witness. Authors did not, and do not, have access to information that could identify individual participants after data collection. (PDF) [file pone.0314702.s001.pdf]

Supplementary Table 1. Hospitals involved in the Familiarisation and Neonatal Sepsis Studies. Subsequently to acquisition of ethical permission, local administrative clearance was secured from each of the 5 participating Ugandan hospitals. Clinical specimens were collected from patients and data was accessed for research purposes between 7 November 2022 and 18 May 2023. All adult participants in FS provided written informed consent. For NSS, parents or guardians provided written informed consent for neonates. For participants who were unable to read or write, consent was obtained in the presence of a witness who read and explained the ethics statement. Participants confirmed their consent by affixing their thumbprints to the consent forms, which were also countersigned by the witness. Authors did not, and do not, have access to information that could identify individual participants after data collection.

**Hospital name**	**Address**	**Hospital level**	**Level of care provided**	**Care System**	**Capacity**	**Study involvement**
Kawempe National Referral Hospital	Kawempe, Kampala, Central Region, Uganda	National Referral Hospital	Maternal care; antenatal, postnatal and immunisation services, newborn care, NICU services	Public	200-bed	FS & NSS
Mulago Specialized Women and Neonatal Hospital	Mulago, Kampala, Uganda	Specialized Women and Neonatal Hospital	Specialized care and services for complex pregnancies or neonates, NICU services, perinatology services	Public	450-bed	FS & NSS
Jinja Regional Referral Hospital	Jinja, Jinja District, Uganda	Regional Referral Hospital	Maternal: antenatal and postnatal care and newborn care	Public	600-bed	FS & NSS
Iganga Hospital	Iganga, Iganga District, Uganda	District Hospital	General maternal and newborn care	Public	100-bed	FS & NSS
Kiwoko Hospital	Kiwoko, Nakaseke District, Central Region, Uganda	Private Community Hospital	Maternal care; antenatal postnatal and immunisation services, abnormal deliveries and referrals newborn care, NICU services	Private	250-bed	NSS only
