# Supplementary material for: Diagnosis of neonatal and adult sepsis using a Serum Amyloid A lateral flow test
Source: PLoS One. 2025 Feb 12;20(2):e0314702. doi: 10.1371/journal.pone.0314702 (PMC11819581; doi:10.1371/journal.pone.0314702)
Supplement: S2 Table — Significance level α = 0.05 was set in all cases and an equal number of patients in each comparison group was required. Resultant Power calculations using G*Power, based on a small result difference (0.2) and power factor 0.8, yielded a study population size n = 620 (n = 310 normals and n = 310 sepsis patients) for the hospital-based study and a population size n = 620 (n = 310 normals and n = 310 sepsis patients) for the field-based study. However, it should be noted that these participant requirement numbers also exceeded those required for medium (0.5) and large (0.8) expected differences at power factor 0.95. This is based on the possibility that a spectrum of SAA levels will be detected in the sepsis groups, compared to low levels in the control groups. Thus, all resultant experimental data was highly powered. (PDF) [file pone.0314702.s002.pdf]

Supplementary Table 2. Calculation of Sample Size N and corresponding test and control and test group numbers*. Significance level α = 0.05 was set in all cases and an equal number of patients in each comparison group was required. Resultant Power calculations using G*Power, based on a small result difference (0.2) and power factor 0.8, yielded a study population size n = 620 (n=310 normals and n=310 sepsis patients) for the hospital-based study and a population size n = 620 (n = 310 normals and n = 310 sepsis patients) for the field-based study. However, it should be noted that these participant requirement numbers also exceeded those required for medium (0.5) and large (0.8) expected differences at power factor 0.95. This is based on the possibility that a spectrum of SAA levels will be detected in the sepsis groups, compared to low levels in the control groups. Thus, all resultant experimental data was highly powered.

**Power->**		**0.8**			**0.95**			**0.8**			**0.95**		
		**One-tailed t-test**			One-tailed t-test			Two-tailed t-test			Two-tailed t-test		
**Expected difference**		**N**	**Control**	**Test**	**N**	**Control**	**Test**	**N**	**Control**	**Test**	**N**	**Control**	**Test**
Large	0.8	**42**			70			52			84		
			**21**	**21**		35	35		26	26		42	42
Medium	0.5	**102**			176			122			210		
			**51**	**51**		88	88		64	64		105	105
Small	0.2	**620**			1084			788			1302		
			**310**	**310**		542	542		394	394		651	651

* As function of exponential power, size of observed differences and t-test tail.
