# Supplementary material for: Diagnosis of neonatal and adult sepsis using a Serum Amyloid A lateral flow test
Source: PLoS One. 2025 Feb 12;20(2):e0314702. doi: 10.1371/journal.pone.0314702 (PMC11819581; doi:10.1371/journal.pone.0314702)
Supplement: S3 Table — SAA lateral flow test resultsa for normal human sera (n = 30) and infected human sera (n = 20) compared to parallel SAA-ELISAb,c quantification of SAA levels. (PDF) [file pone.0314702.s003.pdf]

Supplementary Table 3. SAA lateral flow test results[a] for normal human sera (n = 30) and infected human sera (n = 20) compared to parallel SAA-ELISA[b,c] quantification of SAA levels.

Sample ID	Clinical Interpretation	SAA-LFT lines	ELISA (µg/ml)	Sample ID	Clinical Interpretation	SAA-LFT lines	ELISA (µg/ml)
1	Normal	3	0.3	1	Inflammation	1	>300
2	Normal	3	1.2	2	Inflammation	1	>300
3	Normal	3	1.4	3	Inflammation	1	>300
4	Normal	3	1.9	4	Inflammation	1	>300
5	Normal	3	0.6	5	Inflammation	1	170
6	Normal	3	1	6	Inflammation	1	>300
7	Normal	3	2.2	7	Inflammation	1	67
8	Normal	3	1.2	8	Inflammation	1	>300
9	Normal	3	0.58	9	Inflammation	1	>300
10	Normal	3	2.6	10	Inflammation	1	282
11	Normal	3	2.8	11	Inflammation	1	250
12	Normal	3	3	12	Inflammation	1	>300
13	Normal	3	0.7	13	Inflammation	1	>300
14	Normal	3	0.5	14	Inflammation	1	>300
15	Normal	3	1	15*	Normal	3	9
16	Normal	3	1.9	16	Inflammation	1	>300
17	Normal	3	1.3	17	Inflammation	1	>300
18	Normal	3	0.4	18	Inflammation	1	>300
19	Normal	3	0.9	19	Inflammation	1	>300
20	Normal	3	2.5	20	Inflammation	1	>300
21	Normal	3	0.5				
22	Normal	3	6.6				
23	Normal	3	3.9				
24	Normal	3	0.9				
25	Normal	3	3.7				
26	Normal	3	0.8				
27	Normal	3	1.9				
28	Normal	3	1.7				
29	Normal	3	1.4				
30	Normal	3	0.5				

[a] Lateral flow test interpretation.: 3 lines = Normal (i.e., no inflammation/infection).
[b] SAA normal range by SAA-ELISA (0 - 15 µg/ml).
[c] SAA-ELISA calibrated to the WHO international standard for SAA (Code 92/680).
