# Supplementary material for: Diagnosis of neonatal and adult sepsis using a Serum Amyloid A lateral flow test
Source: PLoS One. 2025 Feb 12;20(2):e0314702. doi: 10.1371/journal.pone.0314702 (PMC11819581; doi:10.1371/journal.pone.0314702)
Supplement: S4 Table — Clinical specimens. (PDF) [file pone.0314702.s004.pdf]

Supplementary Table 4. NeoSep SAA results from the Familiarisation Study. Clinical specimens.

Sample Type	Sample Size n	SAA Positive	SAA Negative
**Neonates**	50	43	7
**Mothers**	175	168	7
**Adults**	278	208	70
