# Supplementary material for: Diagnosis of neonatal and adult sepsis using a Serum Amyloid A lateral flow test
Source: PLoS One. 2025 Feb 12;20(2):e0314702. doi: 10.1371/journal.pone.0314702 (PMC11819581; doi:10.1371/journal.pone.0314702)
Supplement: Attachment — Submitted filename: Response to Reviewers.docx [file pone.0314702.s005.docx]

**RESPONSE TO REVIEWERS**

PONE-D-24-28029
Diagnosis of neonatal and adult sepsis using a Serum Amyloid A lateral flow test.
PLOS ONE

**12 July 2024**

**1. Journal Decision (11 July 2024):** Thank you very much for submitting your manuscript to PLOS ONE, and for responding to our recent requests regarding your submission. After careful evaluation, we have decided that your submission does not meet our publication criteria and must be rejected.

Based on our evaluation of your manuscript and the information you provided, we do not feel that your submission meets our ethical requirements for human subjects research submissions. PLOS ONE requires that research meets all applicable standards for the ethics of experimentation and research integrity (http://journals.plos.org/plosone/s/human-subjects-research). We reserve the right to reject any submission that does not meet our internal ethical standards, which in some cases are more stringent than local ethical standards.

We recognize that you obtained ethical approval from your local ethics committee after you began your study: your ethics approval document from  Maynooth University and Uganda National council of science and technology  is dated  24 June 2022 and 03 October 2022 respectively, while participants' personal data were collected and accessed beginning on 17 June 2022. For human subjects research, PLOS ONE requires that authors obtain ethics committee approval before beginning the study, and we do not accept retrospective ethical approval.

As a result of these concerns we cannot consider the manuscript for publication at PLOS ONE. I am sorry that we cannot be more positive on this occasion, but hope that you understand the reasons for this decision.

**Author response:** I understand your entirely reasonable and correct response above given my incorrect phrasing of dates within the ethical section of the original manuscript and in the response to queries during manuscript submission.

However, ethical approval was actually obtained before work began and work could not have commenced without all ethical approvals. Thus, I can confirm that specimen collection commenced on **7 November 2022**, which is, in accordance with *PLOS ONE* requirements, after the confirmation of:

(i) Makerere University ethical approval (letter dated 17/6/2022 and operational period 17/6/2022-17/6/2023),
(ii) Maynooth University ethical approval (letter dated 24 June 2022 and expiry 30 June 2023) and
(iii) Ugandan National Council for Science and Technology ethical approval (letter dated 3/10/2022) and operational period 3/10/2022 – 3/10/2024).

The revised manuscript clearly illustrates this chronological order of events, the importance of which is now crystal clear to me with view to submission to *PLOS ONE,* as follows:

***‘Ethical approval.*** *Overall project ethical permission was secured at Makerere University School of Public Health (Reference: SPH-2022-220; duration 17 June 2022-17 June 2023) and Maynooth University (Reference: BSRESC-2022-2477046; duration 24 June 2022-30 June 2023), respectively. Subsequently, local administrative clearance was secured from each of the 5 participating Ugandan hospitals. The study was also registered with the Uganda National Council of Science and Technology, registration number HS2439ES (Approval date 3 October 2022; duration 3 October 2022 – 3 October 2024). Clinical specimens were collected from patients and data was accessed for research purposes between 7 November 2022 and 18 May 2023. All adult participants in FS provided written informed consent. For NSS, parents or guardians provided written informed consent for neonates. For participants who were unable to read or write, consent was obtained in the presence of a witness who read and explained the ethics statement. Participants confirmed their consent by affixing their thumbprints to the consent forms, which were also countersigned by the witness. Authors did not, and do not, have access to information that could identify individual participants after data collection.‘*

Furthermore, it will be clear that specimen collection and testing were completed prior to expiration of all requisite ethical permissions. If necessary, excel spreadsheets with all anonymised patient data, specimen collection and testing dates is available for viewing.

Hopefully this resolves all lack of clarity on my behalf relating to dates, and assures *PLOS ONE* of our strict adherence to ethical standards.

Sincerely

Professor Sean Doyle

Professor Peter Waiswa

Joint Corresponding Authors.
