# Supplementary material for: Diagnosis of neonatal and adult sepsis using a Serum Amyloid A lateral flow test
Source: PLoS One. 2025 Feb 12;20(2):e0314702. doi: 10.1371/journal.pone.0314702 (PMC11819581; doi:10.1371/journal.pone.0314702)
Supplement: Attachment — Submitted filename: Response to Reviewers.docx [file pone.0314702.s006.docx]

**Manuscript title: Diagnosis of neonatal and adult sepsis using a Serum Amyloid A lateral flow test.**

**Manuscript number: PONE-D-24-29224R1**

**RESPONSE TO REVIEWERS (11 November 2024)**

Reviewer #1: Reviewer comments
Manuscript title: Diagnosis of neonatal and adult sepsis using a Serum Amyloid A lateral flow test.
Manuscript number: **PONE-D-24-29224R1**

Abstract
1. Page 2, line 38: Author States (AS)….’as a superior biomarker for the diagnosis of neonatal sepsis.’
Reviewer comment (RC): The authors have missed the point which I was trying to make.
The above sentence is required to be framed as …’as a superior biomarker for use in the sepsis screen for neonatal sepsis.’ OR ’….as a superior biomarker for the diagnosis of probable neonatal sepsis.’
**Author response 1:** Done. Text now reads:

*‘However, serum amyloid A (SAA) detection has potential as a superior biomarker for the diagnosis of probable neonatal sepsis.’*

Introduction
1. I thank the authors for their clarification of the queries raised.

The authors response to the undermentioned queries no. 2, 3, 4 from the previous review may be added in a concise form in the introduction, material & methods or discussion section.
2. Why did the authors select SAA over Serum Procalcitonin and CRP?
3. Colorimetric card tests are available for CRP, PCT and are very efficiently being used in field out of hospital settings. Then why SAA?

**Author response 2:** Our pleasure. Reviewer Q2 and Q3 are addressed based on pre-existing text by addition of this sentence in R2 Introduction as follows:

*‘Hence the choice of SAA over PCT and CRP as biomarker, with a location-compatible detection system, for our study.’*

4. In a suspected sepsis patient one would be collecting samples for Complete Blood Counts (CBC) and Blood culture. Thus minimally invasive sampling for SAA wold not be much of a benefit.

**Author response 3:** Noted. We insert the text in yellow in R2 below, which highlights that it is not just lack of centralised testing but availability of ANY testing in resource-limited environments that is the problem, to address Reviewer Q4:

*‘It has the potential to facilitate a rapid clinical decision in the absence of centralised test facilities or any existing patient-side biochemical analyses, and to initiate antibiotic therapy to combat sepsis.’*

Results
The explanation that ‘These 78 neonates were clinically diagnosed with sepsis by clinicians independent of the NeoSep-SAA result.’ Must be adequately be mentioned in the manuscript at a relevant place.

**Author response 4:** Thank you. We have inserted this comment in yellow in Results as follows:

*‘Additionally, 78 neonates within the CCG were clinically diagnosed with sepsis (Confirmed Sepsis: CS). Sepsis diagnosis was based on a suspicion of infection and combination of clinical symptoms or risk factors aided where possible by a CRP test, blood cultures and confirmed by an independent clinician’s diagnosis. Thus, these 78 neonates were clinically diagnosed with sepsis by clinicians independent of the NeoSep-SAA result. Of the 78 neonates, 72 (92%) neonates with a confirmed sepsis diagnosis tested positive in the NeoSep-SAA (****Table 4****).’*

Reviewer #2: Reviewer has addressed all the comments and suggestions from the editor and the reviewers, and I have no additional comments.

**Author response:** Thank you.
